# Jordan and Syrian humanitarian refugees' dilemma: international law perspective

**DOI:** 10.1016/j.heliyon.2022.e09377

**Published:** 2022-05-04

**Authors:** Amir Salameh Al Qaralleh

**Affiliations:** a Department of Political Science, The University of Jordan, Amman, Jordan; b Prince Al Hussein Bin Abdullah II School of International Relations, The University of Jordan, Jordan

**Keywords:** Humanitarian asylum, UNHCR, Refoulment, Syrian refugees' protection, International law obligations, Jordan and Syrian refugees influx, Shared responsibilities, International burden-sharing

## Abstract

Jordan is not a signatory of the 1951 humanitarian refugees' treaty or its 1967 optional protocol. However, Jordan in 1998 signed a Memorandum of Understanding with the UNHCR. Therefore, Jordan is legally obligate to receive refugees. As a result of the 2011 Syrian civil war, hundreds of thousands of refugees fled to Jordan. Syrian refugees have become a dilemma for Jordan, giving UNHCR, donor states, and the international community's failure to fulfill their obligations towards Jordan fully. This article reviews Refugees' protection under the global legal system**.** It informs Jordanian and UNHCR's legal and institutional framework while handling Syrian refugees' issues. It also evaluates Jordan's response to Syrian refugees and the challenges that have faced Jordan since 2011. Finally, the study examines whether or not UNHCR, donor countries, and the international community are committed to Jordan's obligations.

## 1 Introduction

The issue of refugees has become of great importance due to humanitarian commitments that the world is witnessing ever since 1945 to date. After the founding of the United Nations, efforts were exhausted to deal with Europe's humanitarian influx that has been resulted from World War II, which has become a burden on the European community. This influx has become a real tragedy, increasing the number of refugees seeking protection from other countries. The establishment of the United Nations Economic and Social Council in 1945 and the United Nations High Commissioner on refugees Council in 1950 came as a realization of a lofty international vision to cope with humanitarian dilemmas. Those dilemmas arose after World War II and its unforgettable tragedies, including the displacement of millions of Europeans from their homelands. The global efforts coincided with the necessity of codifying international customs, which resulted in the consensus to sign and ratify the Refugee Convention of 1951 and its 1967 optional Protocol. This convention and its protocol have become the legal framework through which the Commissioner exerts its actions globally, and it is binding on signatory states. Therefore, the mechanisms of dealing with the displaced people and humanitarian issues regulated through an international agency responsible for providing protection, care, financial and logistical services to refugees forced to leave their countries either as a result of global or regional wars or civil wars. Accordingly, pose a severe threat to their fundamental freedoms, including the right to movement, work, and expression, in addition to fear of torture, inhuman treatment, or ethnic or religious cleansing.

It is worth noting that asylum is not limited to humanitarian asylum resulting from wars, but includes humanitarian asylum based on personal opinion, belief, gender, political persecution, and asylum that results from natural disasters. Therefore, when the asylum criteria are available and valid for a person who wishes to enter a particular country as a refugee for the aforementioned reasons, and their credibility and eligibility has been confirmed, the host country must deal with him/her as a refugee and grand him/her all the rights stipulated in international conventions, the Universal Declaration of Human Rights, and the two covenants on political, civil, social, cultural, and economic rights (Abed Alshaheed, 2009). Article 2 of the Universal Declaration of Human Rights states that" no one shall be subjected to cruel, inhuman, degrading treatment or punishment". Also, Article 14 of the same Charter gave the inherent right to all individuals to seek asylum in other countries within the aforementioned cases (the Universal Declaration of Human Right, 1948). Therefore, any circumstance in which an individual may believe that he/she is subjected to psychological, physical, or natural persecution based on natural disasters is considered a compelling reason for some individuals to leave their countries of origin and travel to seek asylum in another country.

Accordingly, asylum includes various types, all of which fall within one concept, which is that for each individual, when the criteria for seeking asylum in another country are fulfilled, the host state receives him/her and treats them humanly, which may result in them obtaining permanent residency and then nationality. Hence, the right of asylum is a right that arises directly for a person when his/her rights, fundamental freedoms, and life are endangered or seriously violated (Abed Alshaheed, 2009). Therefore, refugee enjoys the international legal protection stipulated in the 1951 and its 1967 optional protocol. The host country, for example, in the case of political, diplomatic, religious, or social asylum, must respect international legitimacy, and international humanitarian law by adhering to international conventions that regulate the issue of asylum in general. It is worth noting that protected individuals do not have the right to waive any of their rights stipulated in international conventions, nor does the host state has the right to compel them to waive any of these rights (United Nations General Assembly, 1951). Therefore, it is the responsibility of the host country to secure medicine, shelter, food, infrastructure, and services as long as the refugee is present on its territory until either obtain residence in a third country or give them the right to reside permanently. It is not possible in the case of political, social, ideological, or humanitarian asylum to force the refugees to forcibly return to their country of origin, giving that this may endanger their life. In this case, it is necessary to search for a country that welcomes the refugee to reside there permanently.

After the collapse of the former Soviet Union in 1990, international conflicts decreased and, unexpectedly, internal civil wars increased, resulting in instability and mass killing during the last century. This situation led to forced displacement from those countries to pursue safety and shelter. Rwanda, Somalia, and Yugoslavia are only vivid examples of civil war and adverse international peace and security outcomes. At the beginning of 2010, the waves of the so-called Arab Spring began in Tunisia, which then spread to Egypt, then Libya, and later to both Syria and Yemen and, to a limited extent, to some other Arab countries. The rebels demanded radical economic and political reforms then escalated their motive to request immediate overthrow of existing regimes. The Arab Spring in Tunisia, Egypt, Libya, and Yemen was short-lived as the regimes of Zine El Abidine Ben Ali in Tunisia; Hosni Mubarak of Egypt; Muammar Gaddafi in Libya, and Ali Abdullah in Yemen were toppled.

The events in Syria were not the same as other Arab countries giving Russia and China endless support to Assad's regime. Both countries prevented the issuance of the United Nations' resolution authorizing force against the government. The Assad's regime is resisting change and toppling, and it has practiced many illegitimate, immoral, inhuman, and oppressive actions against its people. These situations led to hundreds of thousands of deaths and injury among innocent civilians' people and forced them to displace internally and externally, questing peace, safety, and freedom. Millions of Syrian fled to neighboring countries, namely, Lebanon, Turkey, and Jordan, escaping the regime's brutality. Jordan, since 2011, has received waves of Syrian refugees. Refugees were accommodated in local rural areas, and in a later stage, Jordan situated them in Za'atari and Azraq camps.

Meanwhile, other refugees whom Jordanian families sponsored were allowed to live and resided in Jordanian cities. There is no doubt that Syrian humanitarian asylum in Jordan has exceeded its ability. This situation requires the UNHCR, donor states, the international community's commitment to humanitarian responsibilities and obligations towards Syrian refugees in Jordan.

## 2 Background

Firmly, humanitarian asylum issues have the international community's noticeable concerns. Considerably, it is one of the universally recognized fundamental human rights. Throughout modern history, many societies suffered from forced displacement, which resulted from either direct occupation or ruling regimes' oppression. Since the founding of the United Nations, efforts have intensified to address asylum and refugees' issues. The international body sought to create adequate guarantees for human rights and thus deal with violations practiced against innocent civilians, which may force them to flee their homeland in search of a safe haven. After the end of World War II, a new international legal system emerged to sensitively cope with refugee protection in light of European migration and forcibly displaced movements that pursued stability and safety (Alshoubaki, 2018). The United Nations High Commissioner's Office for Refugees (hereafter UNHCR) was formed to handle the aftermath of the Second World War's Eastern European citizens. Those masses fled their own countries seeking a better life and safe conditions in Western Europe (UNHCR Website). Allegedly, the Commissioner had only three years to accomplish its mission then disband. However, it is still functioning now. The Commissioner aims at aiding and protecting refugees, forcibly displaced people, and stateless individuals. It also seeks to assist refugee's voluntary return, local amalgamation, or resettlement to a third-party country (UNHCR Website).

The UNHCR efforts lie in seeking enduring solutions for refugees, including ordinary and safe return to their homeland, systematically rehabilitating them and guaranteeing their rights in host countries (UNHCR Website; Faselah 2008). The United Nations Refugee Agency is governed by the United Nations General Assembly and United Nations Economic and Social Council. The collaboration between the three bodies aims to efficiently protect refugees' rights and provide better live-standard conditions in host countries (UN, 2016; Batori 2007; Abu Harb 2018).

In 1951 the United Nations General Assembly sponsored the Refugees' Convention with 144 state parties (United Nations General Assembly, 1951). All of these states ratified the treaty, which then became legally binding. The convention's main objective is to protect refugees against forcible return to a country where they might face life - threatening situations or lack of freedom (Ibid). Sixteen years later, the 1951's convention protocol signed by 146 states to address states' cooperation to ensure that refugees' rights are legally protected and respected (United Nations General Assembly, 1967).

Undoubtedly, since the establishment of the United Nations, massive efforts were exhausted to handle refugees' issues through establishing effective and comprehensive international refugee laws. However, host states vary in their response to humanitarian refugees. Accordingly, there are numerous commitments and legal obligations that states resort to when handling refugees' matters. Jordan's humanitarian efforts to welcoming refugees are based on local and regional laws and collaborate with international humanitarian organizations to receive and manage humanitarian asylum seekers' situations (Alshoubaki, 2018). As a result of humanitarian displacement and host countries' receptionist of refugees, hardship begins, which entails additional economic, demographic, and social burdens on the host country's side. Jordan, since 1948, has dealt with waves of humanitarian asylum resulting from setbacks, coincided with the massive displacement of Palestinians, which occurred in 1967 as a result of the occupation of the West Bank lands by Israel. Then, after the second Gulf War of 1991, a significant number of Iraqis fled to Jordan, and the scene repeated after the fall of Baghdad and the occupation of Iraq in 2003. Meanwhile, and as a result of the bloody events in Syria in 2011, waves of humanitarian asylum began to cross into Jordan, whose numbers till the moment exceeded nearly a million. These events posed a significant challenge to Jordan, the latest of the Syrian displacement, which negatively affected the economy and social structures.

The so-called Arab Spring waves broke out in 2010. The Syrian Spring emerged in March of 2011, which escalated to a large - scale heinous civil war. The primary reason for this civil war was some students' torture, who painted anti-government graffiti. Consequently, a political uprising started. The anti-government protests grew steadily across Syria, and suddenly, tens of thousands of Syrians demanded immediate extensive and comprehensive political and economic reforms, then escalated to request Assad's resignation (Al Qaralleh, 2018; Al Qaralleh and Albarasneh, 2019). The United Nations described the Syrian crisis as the 21^st^ century's worst humanitarian horror, that deeply tested the international community's accountability and capacity while responding to protect civilians by easing their suffering and fostering the resilience of host communities and Syrian refugees (BBC News, 2020; Munch, 2020). According to an estimate by UNHCR (2020), more than 400,000 people lost their lives since the beginning of the Syrian civil war, 5.6 million forcibly fled the country, and almost 6 million have been internally displaced (Ibid). Out of 5.6 million who fled Syria, Jordan has provided refuge to nearly 1.36 million. Amman and Northern governorates of Jordan hosted 90% of the total refugees, while 10% living in camps (Jordan Response Plan, 2020). The UNHCR's data shows that only 662,790 registered in Amman's regional office (UNHCR Operational Portal, 2021).

Jordan is always committed to its moral attitudes towards refugees' issues. In so doing, Jordan exceeds its capabilities and deals with Syrian refugees within the short- and long-term needs, which significantly strain the country's powers. Compared to the population of Jordan, the percentage of Syrian refugees is 15%. Consequently, Jordan is the second host country worldwide (Jordan Response Plan, 2020). This fact shows the great efforts exhausted towards refugees within Jordanian territories. The influx of Syrian refugees into Jordan has a substantial impact on government institutions and negatively affected the state's infrastructure ability to absorb massive population inflation. In 2016, the World Bank issued a report that estimates Syrian refugees' annual cost. Each refugee cost the Jordan government almost $3,750 (Jordan Response Plan, 2020). The influx of Syrian refugees exceeded one million, which means that the yearly cost to host Syrian refugees is over $ 3.75 billion (The Jordan Times, 2016).

In 2017 the Jordanian government indicated that since the Syrian crisis outbreak in 2011 until 2017, it is estimated that refugees' existence in Jordan cost the state budget almost $10 billion (Russia Today, 2017). Giving the above mentioned, the successive Jordanian governments showed bad reactions to adopted open- border policy, which also combined with the scarcity of limited resources and limited government public spending (Sharp, 2016). Citizens of host countries have primarily represented refugees as passive recipients who drain resources or deplete natural resources (Phililps, 2003). To a certain extent, Jordanians believe that Syrian refugees negatively impacted the fragile economy, harmed their living standards, and shrank their working chances.

This article reviews Refugees' protection under the international legal system**.** It informs Jordanian and UNHCR's legal and institutional framework while handling Syrian refugees' issues and matters. It also evaluates Jordan's response to Syrian refugees and the challenges that have faced Jordan since 2011. Finally, the study examines whether or not UNHCR, donor countries, and the international community are committed to Jordan's obligations.

## 3 Methods

Legal and descriptive- analytical approaches were employed to answer the study's central question related to the difficulties that Jordan faced, and still is, during its reception of Syrian humanitarian asylum, and the repercussions of international failures in helping Jordan in its quest to secure safe and decent asylum for the Syrians. Accordingly, all international legal treaties, agreements, and texts related to humanitarian asylum were recalled and described, and then analyzed and reflected on the reality of Syrian humanitarian asylum in Jordan. in the same context, the Jordanian domestic legal texts regulating humanitarian asylum were also located and analyzed accordingly. Hence, the Memorandum of Understanding signed between Jordan and UNHCR in 1998 also was analyzed to uncover whether Jordan is obligated to receive humanitarian asylum.

In a related matter, the responsibility that legally falls on the shoulders of the UNHCR, the donor states, and the international community, was analyzed to reveal collective international efforts to fulfill obligations towards Jordan's reception of Syrian humanitarian's influx. The pledges made by both the UNHCR, and donor states at the beginning of the Syrian asylum to Jordan, and the waves of continuous asylum into Jordan until now, were analyzed. Also, closely related is examining the Jordanian Response Plan (RP) which aims at laying out Jordan's financial, logistical, and infrastructure needs while stations and secure haven for Syrian refugees on its territories. Also, the article reviews the overall economic and financial difficulties that stand in the way of Jordan to carry out its moral and humanitarian duties towards Syrian's refugees', in light of the scarcity of resources, which may force Jordan to resort to a closed-border policy or refoulment of Syrian's refugees' which may result in a regional humanitarian disaster.

It is worth mentioning, that the researcher also relied on previous related literature that dealt with the phenomenon of humanitarian asylum. Meanwhile, the article relied on Jordanian previous literature and resources that dealt with the outcome of Syrian's asylum influx into Jordan and the negative consequence on the state's resources and dilapidated economy that suffers from the scarcity of resources, weak financial capabilities, and lack of infrastructures necessary for the sustainability of asylum and its relationship to the failure of UNHCR, and donor states to ideally fulfill their financial obligations associated with the Syrian's humanitarian asylum in Jordan.

## 4 Results and discussion

### 4.1 Refugees' protection under the international legal system

The growth of the humanitarian asylum phenomenon is only an inevitable consequence of denying individuals, groups, and people's rights. Defending refugees' rights has become the focus of the global community's attention, aiming to guarantee and protect human rights and face possible denial or violations. If human rights are central international issues, humanitarian asylum is of particular importance, giving the increasing proliferation of rights violations of individuals and groups, resulting from the expansion of power struggles' hotbeds and regional internal conflicts and wars. The outcome of this unrest is the displacement of millions of people and their influx into neighboring countries seeking safe asylum.

To certain extent, the humanitarian refugees' regime is new. It was legally created immediately after the end of World War II. This resulted from the influx of more than 40 million Europeans due to the post-war era, which directed the international community to think seriously to establish a legal system in which refugees are protected from potential risks that they may face in displacement's countries (Al-Marsad, 2016). Theoretically and practically, states, according to international law, are responsible for protecting the human rights of their citizens and aliens within their territories (Ibid:7). Article 2 of the 1948 Universal Declaration of Human Rights indicates that no person shall be subject to torture, punishment, cruel, brutal, or degrading treatments (The Universal Declaration of Human Rights, 1948, Universal Declaration of Human Rights, 1948: Art 2). Article 14 of the declaration gave individuals the inherent right to seek asylum in other countries in persecution cases (Ibid: Art 14).

In some circumstances, states may fail in their duties, either because of inability or unwillingness to protect citizens and residents on their lands. In these situations, individuals who fare that their human rights are under grave violations flee forcibly or voluntarily their homeland seeking security and safety. In this case, protecting innocent civilians escalated to the international community, ensuring that fundamental rights are maintained and protected. Protecting the rights of displaced people is the international law body's responsibility, which constitutes the international refugees' protection regime. This regime deals with civil war and ethnic conflicts, and related humanitarian asylum issues (Hathaway, 1984). This situation necessitated the urgent need to establish the United Nations High Commissioner for refugees in 1950 to provide international protection for refugees and seek robust solutions to their suffering. On December 14^th^ 1950, the UN General Assembly endorsed UNHCR's statute and started to function until now (UNHCR Website). Worth noting that 144 states signed and ratified the 1951 convention. The same states signed 1967's protocol in addition to two new members. The total number of state parties to either treaties or both is 149 (UNHCR Website).

The 1951's refugee convention and its 1967's optional protocol are the legal basis that defines standards to deal with refugees' issues globally. The core principle of the 1951 convention is non-refoulment, which emphasizes forbidding enforcing refugee to return to a country in cases where he/she might face severe life-threatening circumstances or constraining personal freedom (UNHCR Website). The non-refoulment principle reflects the international community's commitment to ensuring that individuals should enjoy their fundamental rights, including the right to life, not to be subjected to torture, cruel, inhuman, or degrading treatment. The Geneva Convention Relating to the Status of Refugees and the United Nations Convention against Torture and other Cruel, Inhuman or Degrading Treatment or Punishment prohibits refoulment of refugees or people at grave risk if forcibly deported to their countries. Article 33 of the refugee convention stipulates the illegality of refoulment, giving endangerment of a person's life or the possibility of torture on race, religion, or political preferences. Likewise, Article 3 of the convention against torture states that a refugee may not forcibly be expelled or returned to his country, as this could pose a risk of torture or assassination (Refugee Convention, 1951; United Nations Convention Against Torture, 1984; Allain, 2001; Chan, 2006; Al-Marsad, 2016).

The convention and its protocol represent the comprehensive international mechanism that secures refugees' fundamental rights protection. The convention and its protocol regulate refugees' conditions within host states (Abed Alshaheed, 2009). The UNHCR is making strenuous efforts to support the international protection regime manifested in 1951's convention and its protocol. The Commissioner's core refugees' objective is to ensure governments adhere to international refugees' law, preventing displacement and meeting vulnerable people's needs (Abed Al Hameed, 1987; Alwan, 2004; Abu Al-Wafa, 2006; Jwelly, 2005).

One of the UNHCR's primary duties is to ensure the recognition of refugees' legal status, granting their refuge and safeguarding the respect of fundamental human rights under international standards. To achieve this, the UNHCR operates in countries of displacement along with host states (UNHCR Website). According to the 1951 refugee convention, a refugee is a person who suffers from the condition of fear associated with persecution based either on race, religion, nationality, being a member of a specific political or social group, or due to political opinions and attitudes. In the same context, the refugee must be outside his country of origin and not wish to return due to fear for his/her life and lack of public freedom (Refugee Convention, 1951).

According to the 1951 refugee convention, the host country is the one that has, in principle, the responsibility to protect the refugees on its territory (Refugee Convention, 1951). Hence, the signatories to the convention and protocol are obligated to implement its provisions (Abed Alshaheed, 2009; Alshoubaki and Harris, 2018; Alshoubaki, 2018). Articles 3–11 of the convention contain requirements that oblige state parties not to discriminate between refugees based on race, religion, or nationality and grant them care equivalent to their citizens. Consequently, they are entitled to practice their religious rites, respect their way of raising children, and treating them the same as foreigners residing on their territory (Refugee Convention, 1951). While Articles 12–16 deal with refugee law. Articles 17–19 specify refugees' rights to gain employment. Articles 20–24 relate to the right to shelter, education, social security benefits, government relief, and health care. Refugees' right to movement is indicated in Article 26. While Articles 27 and 29 address the right to obtain identity cards and travel documents. Article 33 prohibits deportation forcibly or expelling refugees to their country of origin, which may pose a life-threatening situation (Refugee Convention, 1951).

It is worth noting that the 1951 convention did not explicitly stipulate refugees' duties while being in a host state. The convention only pointed out that refugees must obey the receptionist state's laws and internal regulations. Meanwhile, refugees should abide by measures to maintain public order in the host state. Whereas, the 1969 African Union's Organization of African Unity Convention (OAU) stipulated that refugees are prohibited from harming the relationships between member states through actions lead to tensions or outbreak between signatory countries. Meanwhile, refugees are prohibited from employing actual weapons or media outlets against host state (The Organization of African Unity Convention, 1969).

Dealing with humanitarian asylum issues requires global awareness to balance international humanitarian law and grave responsibilities that fall on host states. Given this, it must be taken into account that the burden does not fall on the host country alone but on the UNHCR and the donor countries, respectively. It is stated in the 1951 convention's preamble that granting the right to asylum places massive burdens on receptionists' states. Hence, this requires the urgent need for international cooperation to deal with refugees' problems (Refugee Convention, 1951). In this essence, the country that receives large numbers of the humanitarian influx has the right to obtain financial assistance to face excessive financial burdens that entail high numbers of human groups on its territory. This assistance should be provided by the Commission and the donor states, giving their presumable belief in the humanitarian and social nature of refugees' cases. What makes the host country's task very difficult is Article 20 of the 1951 convention which, stressed the necessity for the host states to treat refugees as it treats its citizens. The treatment includes providing food, medicine, education, health care, etc.

Logistically, the host country's financial support needs are determined in light of refugees' numbers on its territory. However, the obstacle is that what the host country requests in terms of financial aid may not be fully available. The Commissioner's activities and responsibilities are multiple and comprehensive, which requires the necessity to distribute its efforts and capabilities worldwide. Therefore, the support that the host country may receive depends on its designed plans and programs to deal with similar refugee cases (Abed Alshaheed, 2009; Karns et al., 2017; Reuters, 2015). In return for refugees' presence on its territory, the host state has the inherent right to preserve its political entity, stability, security, and cohesion of its social structure. The host country has the right to deny entrance of individuals who committed a serious war crime, crimes against humanity, ethnic cleansing, and aggression crimes, or persons who committed a grave crime before they entered the receptionist state. Host countries also have the right to enforce work, education, freedom, mobility curfews to either control the flow of significant numbers of refugees or control and contain the spread of dangerous pandemics.

While refugees are situated on the host state's territory, the host state is obligated to treat refugees similar to the treatment of foreigners residing on its territory. It must also adhere to any international agreements beneficial to advance refugee's circumstances. In the same context, the host country must consider respecting refugees acquired related to personal status, including, but not limited to, marriage, divorce, and inheritance. Recognition of the refugees' right to litigate before local courts is also a right that the host country must abide by and support the refugees to obtain residency and then naturalization. Host countries also should seriously encourage the voluntary return to the land of origin or seeking resettlement in third - party countries. In the same vein, the host country should not discriminate between refugees settled on its territory. It should also respect religious feelings and refrain from punishing refugees for illegal entry into its territories (Abed Alshaheed, 2009; Da Costa, 2006; Bjørkhaug, 2020; Refugee Convention, 1951).

One of essential principles of refugees' legal regime is the burden - sharing concept, which UNHCR has defined as an integral part of international cooperation. Hence, states bear the responsibility for refugees who entering neighboring countries (UNHCR Website). The preamble of the 1951 refugee convention indicated this principle by noting that granting humanitarian asylum by certain countries entails heavy burdens on them. This inevitably requires international cooperation to deal with humanitarian asylum worldwide (Refugee Convention, 1951). Precisely, the burden-sharing principle is formulated to deal with the massive influx of refugees into the receptionist state. This influx, in most cases, poses a heavy and complicated burden on asylum-receiving countries (Volker and Garlick, 2016). UNHCR has continuously and diligently indicated that procedures related to burden-sharing include financial assistance as well as adequate physical protection of refugees. The common perception is that humanitarian asylum issues are nothing but evidence of the international community's anxiety to adhere to solidarity and share responsibilities (Boswell, 2003; Al-Marsad, 2016). It is imperative to emphasize that burden-sharing does not mean denying abiding refugees' duties and responsibilities by 1951 signatory states. Rather, its implications are related to the size of refugees, which exceeds host state's capacity. Hence, this inevitably requires the international community to intervene to share humanitarian asylum burden (Volker and Garlick, 2016).

The overall international understanding is that burden-sharing principles constitute a legal obligation. However, the extent of its binding authority is a subject of great debate. The commissioner sought to apply burden-sharing on 1951 signatory and non- signatory states strictly. However, the results were disappointing and unsatisfactory. Denial and betrayal reactions associated with global integration failure to handle the Syrian crisis dominated the scene. The painful forced displacement resulting from Syrian internal unrest is nothing but evidence of the international community's inability to assume its typical responsibilities related to humanitarian asylum's burden.

### 4.2 What governs Jordan's receptionist of humanitarian refugees?

Geographically and historically, Jordan is positioned at the crossroads of a region in turmoil. It has a long record of providing asylum to victimized innocent people (Carnegie endowment for international peace, 2015, Carnegie Middle East, 2015). Throughout the 19^th^, 20^th^, and 21^st^ centuries, Jordan has welcomed multiple refugee waves, mainly Palestinian, Iraqi, and recently Syrians (Alshoubaki and Harris, 2018; Carnegie endowment for international peace, 2015, Carnegie Middle East, 2015). During the Nineteenth Century, resulting from Russian expansion, Circassians fled their homeland and situated in what was then Transjordan, a part of the Ottoman empire. Armenians refugees' wave arrived during World War I and were stationed in Transjordan (WANA Institute, 2019). Adverse outcomes of the 1948 war between Arabs and Israel and the 1967 war were massive waves of refugees who fled Palestine into Jordan searching for shelter and safety. Fewer Palestinian refugees' waves occurred in 1987 from Intifada (Alshoubaki, 2018). Another inflow occurred after the Iraqi's government invasion of Kuwait in 1991, as many Palestinians were forced to leave Kuwait and some other Gulf states (Becker, 2013). After the collapse of Saddam Hussein's regime, many Iraqis fled to Jordan, searching for security and stability. Some refugees who fled to Jordan "emigrated, and many remained in Jordan, building lives and communities over multiple generations" (WANA Institute, 2019:6). Syrian refugees who entered Jordan from 2011 until now are either staying in refugee camps or urban- rural areas or are allowed to enter other cities, such as the capital Amman if they fulfill the terms of sponsorship guarantee of Jordanians citizens (Alshoubaki, 2018). There are three Syrian refugees camps Za'atari, Azraq, and Emirati- Jordanian (UNHCR, 2020).

Jordan never signed the 1951 Refugee Convention or its 1967 optional Protocol (Alshoubaki, 2018; BoJoma'h; 2019, Sadek, 2013), giving the deep Jordanian belief that Palestinians have the inherent right to return to their homeland that the Zionists had occupied in 1948. Meanwhile, there is no Jordanian national legislation or law regulating the mechanism of dealing with refugees' influx or handling legal status while entering Jordanian lands. Despite this, Article 21 of the 1952 Jordanian Constitution prohibits the extradition of political refugees who fled their countries due to persecution on the grounds of their political beliefs or defending freedom (Jordan Constitution, 1952). In 1973 Jordan regulated political asylum's status. Law No. 24 of 1973 Residence and Foreigners' Affairs' stressed that individuals entering the country as political asylum seekers must appear in front of the nearest police station within a maximum of 48 h of entry. Article 31 of the same Law gave powers to the Minister of Interior to determine each case's eligibility, either granting the right to stay or deportation (Law No. 24 of 1973). It is worth noting that Article 31 did not specify what conditions a political asylum's seeker should have that qualify him/her to obtain asylum status, nor did it address the imposition of penalties on asylum seekers who entered the territories illegally (Sadek, 2013). Accordingly, the Interior Minister has broad discretionary powers that allow him to deal with political asylum cases according to his understanding of the asylum seeker's situation.

One of the most critical provisions stipulated in Law No. 24 is that a refugee does not automatically receive residency, work, education, or healthcare. In the same context, a foreigner cannot live in Jordan not unless obtaining a residency permit, which entitles him/her to life for one year, provided that should renew it periodically, and is subject to the Ministry of Interior's approval (Law No. 24 of 1973; Sadek, 2013). Jordan's unwillingness to concede to the 1951 refugee convention essentially does not mean non-compliance with international law legitimacy. This understanding drove Jordan, morally, to sign a Memorandum of Understating (MoU) with UNHCR in 1998. It was bilaterally agreed to enable the commission to carry out its work in Jordan. The Memorandum also defined refugee's legal framework. It has been legally approved allowing all refugees to enter Jordan without discrimination, requiring a visa or residency permit. A grace period of six months was agreed upon, with the Commissioner's pledging to find a country for their resettlement (Memorandum of Understanding Between the Government of Jordan and UNHCR, 1998; UNHCR Global Appeal, 2015; Sadek, 2013; Alshoubaki, 2018).

The 1998 MoU allowed the UNHCR to implement its mandate in Jordan, securing international protection for refugees falling with its jurisdiction. The Memorandum of Understanding became the legal framework through which the Commissioner conducts its affairs in Jordan. It has been agreed on Jordan's acceptance of the 1951 refugee convention's definition, its commitment to respect the principles of non-refoulment, and commitment to treating refugees according to accepted international standards. Among what was agreed upon between Jordan and the UNHCR, related to rights and privileges, that refugees enjoy the freedom to practice religious rites, the right to provide religious education to their children, non-discrimination, and the right to litigate in front of domestic courts. Privileges include exemption from fines for overstaying and fees for leaving Jordanian lands. The UNHCR was also allowed to conduct interviews with asylum seekers in Jordan who had entered illegally and decide their stay within a week. In exceptional cases, when the situation requires multiple measures, the decision must be taken within a period not exceeding a month (Memorandum of Understanding between the Government of Jordan and UNHCR, 1998; Library of Congress Website).

In 2014, two amendments were made to MoU, extending refugees' application processing period from 30 to 90 days and extending refugees' cards from six months to one year (Malkawi, 2014). Despite Jordanian efforts to welcome and aid refugees, it publicly avoids official recognition of refugees under its domestic laws and refer to Arab refugees' waves as Arab brothers; irregular guests; and visitors. This position has no legislature or legal meaning under Jordanian domestic laws (International Labour Organization, 2015). Although Jordan allows Syrian refugees to benefit from health services, food aid, financial assistance, and teaching their children in public schools free of charge, it is not permitted to work (Fakih and Ibrahim, 2015).

Alongside signing the MoU with UNHCR in 2014, Jordan is also committed to the 1994 Arab Charter on Human Rights, which is entirely consistent with the 1951 refugee convention. The Charter stresses the imperative of adherence to fundamental human rights, including the right to litigation, freedom of mobility, freedom of education, and belief and all rights that guarantee preserving human dignity and independence. Therefore, Jordan, being an Arab state that is a member of the Arab League, should grant asylum to people who are forced to leave their countries on the grounds of their race, religion, color, and religious beliefs. According to the Arab Charter on Human Rights, Jordan must fully commit to narrowing the scope of forcible return of refugees (Arab Charter on Human Rights, 1994).

### 4.3 Syrian refugees' institutional framework in Jordan

According to authorities and specialization assigned to it, the Jordanian Ministry of Interior (JMOI), in partnership with its security services, is primarily responsible for ensuring Jordanian national security. Accordingly, the Ministry registers the refugees to determine their eligibility to enter Jordan. It conducts criminal background security checks and then issues identification cards to eligible refugees. Identification cards are used outside the camps to benefit from education and healthcare services. As for camps' refugees, the identity card's use is only to register in Jordan's records as a country of humanitarian asylum. The JMOI cooperates with UNHCR to manage the camps, identify refugees' status, and deal with resettlement cases (Jordanian Ministry of Interior Website; Alshoubaki, 2018; UNHCR, 1998; UNHCR Regional overview, 2015; AlDleffe, 2013).

At the beginning of the Syrian influx into Jordan, the government established a technical committee to discuss refugees' conditions and their flow across the borders. The Jordanian Council of Ministers decided that the Armed Forces, Security Services branches, and all relevant ministries are responsible for managing Syrian refugees' affairs. The mission was designed to deal with refugees crossing the borders and then accommodating them in camps and centers. In a short period, a higher committee was formed to manage the refugees' massive influx to Jordan, headed by the Minister of Interior and the membership of the Ministers of Foreign Affairs, Health, Director of the General Intelligence, Director of Public Security, Director of Gendarmerie, Director of Civil Defense and General Command of the Jordanian Armed Forces. Meanwhile, the UNHCR is responsible for registering the refugees, conducting interviews and, checking their eligibility status (AlDleffe, 2013; Al Sarhan, 2017).

In 2013, the Jordanian Council of Ministers decided to create the Syrian Refugee Affairs Administration, which is affiliated with the Interior Ministry. The administration was established to take care of Syrian refugees' affairs in all Kingdom's governorates. The administration aims to improve the level of services provided to Syrian refugees, and cooperate with Jordanian government agencies and international governmental organizations and NGOs specializing in providing humanitarian, security, food, and relief services. Year after, the administration's jurisdictions were expanded to include Syrian refugees in Jordan, whether inside or outside the camps. The administration name was accordingly changed to the Directorate of Syrian Affairs. It now has overlaps to deal with the right of voluntary return, residency, and resettlement (Al Sarhan, 2017; Al rai daily Newspaper, 2014).

The Council of Ministers, in 2014, decided to appoint the Jordan Hashemite Charity Organization to be the only entity authorized to receive in-kind aid and distribute it to Syrian refugees in Jordan. Thus, non- profit organization was allowed to receive aids from local and international associations, coordinates and supervise its distribution, and oversees Syrian refugee assistance programs. The organization also works side by side with the UNHCR through a joint effort to create a decent life for Syrian refugees' camps in Jordan (Jordan Hashemite Charity Organization's Website, 2014; Al rai daily Newspaper, 2014).

Since 2015, through the Ministry of Planning and International Cooperation (MOPIC, 2020), Jordan issues a national response plan (JRP) to deal with Syrian refugees. The plan led by the Jordanian government provides a proper model for long-term Jordanian partnership between the host country and the international community. Jordan maintains cooperative and transparent principles to develop a mechanism for implementing the plan on the ground through its cooperation with more than 150 national and international partners. Currently, JRP is the only plan that serves the Syrian refugees and vulnerable Jordanians impacted by the Syrian crisis and the consequent waves of asylum towards Jordan. Because of the increase in the number of refugees, its adverse impacts on the electricity and water sectors cannot be mitigated. Therefore, the plan deals with the comprehensive effects of Syrian refugees in Jordan, thus controlling the crisis's burden as much as possible (MOPIC, 2020).

The Ministry of Planning and International Cooperation plays a central role in managing refugees' issues in Jordan. It coordinates the work of NGOs operating in Jordan in their humanitarian efforts related to aiding Syrian refugees. Accordingly, any NGO wishing to assist Syrian refugees must submit an official request to the Ministry to present its proposed program. Through this, these NGOs work is integrated, thus distributing humanitarian efforts according to the organization's capacity and Syrian refugees' needs. The Jordan Ministerial team is cooperating with NGOs to deal with the massive Syrian influx into Jordan (Alshoubaki, 2018; MOPIC, 2015).

The MOPIC's role is designed to divide and organize the work of relevant Ministries, each according to its tasks, which include educational, health, judicial, environmental, food security, empowerment, local governance, shelters, social protection, transportation, and municipal services. Therefore, the Ministry prepares general policies to deal with refugees' crisis and provides the necessary support comprehensively. The General Secretariat of Public Policies manages and monitors the asylum-related issues through the asylum information system and hence assigns an appropriate budget which depends on the Jordanian government, the United Nations' Commissioner, and donor countries. This procedure determines the Jordanian Response Plan. Meanwhile, it locates obstacles and difficulties facing implementation in light of national capabilities' scarcity, national economy weakness, lack of financial resources that Jordan obtains from the Refugees' Commissioner and the international community, and particularly donor states (MOPIC, 2020; MOPIC, 2015; Alshoubaki, 2018).

In addition to Jordanian efforts to deal with Syrian refugees, the UNHCR has a central humanitarian role in Jordan. The UNHCR implements its established goals and principles related to providing protection, social and economic well-being for refugees, and striving severely and purposefully, towards the urgent need for refugee safe resettlement globally. According to the Memorandum of Understanding (1998) signed between Jordan and UNHCR, the former is obligated to resettle refugees within a maximum period of six months, in addition to determining the legal status of refugees. UNHCR mandate also includes providing the highest protection levels for children, women, and the elderly, meanwhile combating gender- based violence (UN Women, 2013). In the same context, the Commissioner cooperates with international and NGOs to provide food, water, shelter, and medical services. UNHCR closely monitors and verifies forced deportation cases through cooperation with Jordanian government agencies to determine refoulment reasons. It also monitors the refugees' movement in-and-out camps movements to protect women, and children from human trafficking and exploitation. To carry out its work, the UNHCR cooperates with the UN Office for Project Services, the World Food Programme, the UN Entity for Gender, and the Empowerment of women and the UN Children's Fund (UNHCR Website; Alshoubaki, 2018; MOPIC, 2015).

### 4.4 Jordan's response to Syrian refugees' influx and its challenges

The influx of Syrian refugees into Jordan since 2011 has driven Jordan to have a strong awareness of the crucial need to create a coordinated approach to tackling Syrian refugees' needs. This promoted the establishment of the Jordanian Response Plan (JRP, 2020) for the years 2014–15, which developed and continued until date. Under the Jordanian government directive, the response plan revolved around collective and participatory efforts that include Jordanian authorities, UN, donors, international organizations, and Local and international NOG's. According to JRP (2020), the juxtaposition of actions is only deepening because the humanitarian response can only survive when the response is flexible, rapid, and targeted (MOPIC, 2020).

In its recent JRP for years 2020–22, the Ministry of Planning and International Cooperation emphasized Jordan's total commitment to global refugees' standards. It indicated that the new plan's structure came to meet the needs of Syrian refugees and vulnerable Jordanians affected by the adverse outcomes of the Syrian crisis. Accordingly, a coordinated and comprehensive response has been formulated to cover all requirements including refugee support, resilience, and an adequate budget. The plan, according to MOPIC, is a pioneering model for responding to humanitarian crises. The plan is for three years and is updated annually to deal with developments. In its preamble, the plan stresses that the objectives include designing targeted programs more relevant to Syrian refugees' needs and reaching the most vulnerable groups. Meanwhile, contribute to enhance the beneficiaries and related systems' capacity, ensuring Syrian refugees' protection, meeting the needs of Jordanians who have been affected by the Syrian crisis consequences, and supporting the Jordanian national systems to maintain quality services provided to refugees (MOPIC, 2020).

Since the beginning of the Syrian crisis in Jordan, government efforts have intensified to provide public services to approximately 655,435 Syrian refugees registered with UNHCR, most of whom are situated in the Kingdom's Northern regions. General services include waste collection and water distribution, environmental management, energy distribution, and transportation. According to a report issued by MOPIC in 2018, the challenges facing the Kingdom's governorates were compounded due to the unproportionate increase in the population, which was 13% resulting from Syrian displacement. This promoted the urgent need to increase the speed of providing public services and strive to develop local communities, given the scarcity of available national resources. The continued pressure on municipalities, accompanied by the need to respond quickly to provide public transport services, solid waste management, energy supplies at reasonable prices, and support social cohesion, overburdened the limited budget. Hence, sustaining vital sectors that serve the Jordanian citizens became a complicated target (MOPIC, 2020; Alrai Daily Newspaper, 2016; Petra Jordanian News Agency, 2016).

As for the health services, since 2019, Syrian refugees have been able to obtain healthcare in governmental hospitals and health centers with a discount rate of 80%, which is equivalent to the non-insured Jordanians' rate. This measure has been adopted so that Syrian refugees can obtain healthcare packages that improve their health status (MOPIC, 2020). The continuation of the Syrian crisis for many years, in addition to the Coronavirus pandemic's repercussions, has exhausted the Jordanian health system (JMoH Website, 2020). Jordan's treatment of Syrian refugees while conducting coronavirus tests and providing immunomodulators and therapy for advanced cases is equivalent to Jordanians. This also called for the urgency to minimize the virus's risk and mitigate spread among Syrian camps. The same procedures apply to Syrian outside the camps (JMoH Website, 2020). The increase of non-infectious and infectious numbers among refugee women, children, disabled, and injured has posed significant challenges for Jordan. The need to ensure proper nutrition for mothers and their infants cannot be ignored, requiring doses against tetanus, and other doses taken by newborns up to two years. The inability to deal with these requirements may entail public health risks and concerns regarding protecting women and newborns from tetanus (MOPIC, 2020).

Children in Jordan enjoy rights to survival, education, protection, healthcare, and development, which the Jordanian Constitution of 1952 guarantees. Meanwhile, Jordan is a signatory of the United Nations Convention on the Rights of the Child (1989). One of the Ministry of Education's central objectives is to improve access to education, equity, and quality for all children (MoE Website). Therefore, since the beginning of the Syrian influx, an immediate response was made to provide free compulsory education for all Syrian children registered with UNHCR (MOPIC, 2020; MoE website; Al Ghribeah, 2017). Accordingly, the MoE established schools in the camps and allowed Syrian refugees' children, whom they are the camps, to attend public schools. To deal with the limited number of schools, a two-shift system – morning and evening; has been employed in public schools that witnessed Syrian refugees' turnout. This was implemented to accommodate Jordanian and Syrian students simultaneously (MoE website; Al Ghribeah, 2017). Despite the Jordanian educational system's ability to deal with the massive Syrian influx and the necessity to provide essential education for refugee children outside the camps, the Syrian asylum has inevitably affected the availability of educational opportunities, equality, and competence. The increasing demand for education has led to maintenance, increasing teachers' numbers, and academic outcomes' challenges. Adopting a two-shift system negatively impacted compulsory schooling outcomes, resulting from reducing teaching hours (MoE website; MOPIC, 2020; Jordanian Department of Statistics, 2019).

At the beginning of the Syrian refugee crisis, Jordan coordinated with UNHCR to deal with the humanitarian influx. Accordingly, illegal refugees were received in shelters in Mafraq city. At a later stage, the Za'atari camp was established in Mafraq with a capacity of 89,000 refugees (Al Wazni, 2012). The influx of Syrian refugees has severely affected the Jordanian housing market, especially in Northern Governorates, which host excessive numbers of displaced Syrians. In Mafraq, apartments and monthly rent prices have been inflated in an unprecedented way. The negative impact was that Jordanian citizens, particularly in Mafraq, were no longer able to pay the high monthly rent, increasing the demand for shelter from Syrian refugees. There is no doubt that the 123,000 Syrian refugees who live permanently in Za'atari and Azraq camps suffer from unsuitable living conditions, especially in the winter season. This, in turn, exhausts Jordan and its ability to sustain and maintain the camps (MOPIC, 2020).

Jordan faced a demographic challenge resulting from successive Palestinian migrations, Iraqi and recently Syrian refugees. Jordan's population has dramatically increased from almost six Million in 2012 to nearly 11 Million to date. Security threat may be formed as a result of the overlap of a group of factors, including the existence of a large gap between the population and the limited available resources, the possibility of extremist ideology among refugees, and border threat, and the possibility of irregular warfare groups entry into Jordan. Population unexpected increase, in the Jordanian case, posed undesired pressure on economic, educational health, and service sectors, which in turn negatively affected popular satisfaction levels and form anger against successive governments, which may lead to future internal instability (Al Sheeb and Nasree, 2017; Carnegie endowment for international peace, 2015, Carnegie Middle East, 2015; Middle East Studies Center, 2015).

Jordan is Third World developing country, as it suffers from natural resource scarcity and thus depends, obviously, on foreign aid to achieve required development, political stability and respond to popular demands. Therefore, the Jordanian economy is weak in the face of external and internal shocks. The Jordanian economy has suffered from the 2008 Global Financial Crisis, which resulted in a significant decrease in foreign capital inflows. The so-called Arab Spring also led to a regional recession, which directly impacted Jordan. The waves of Arab Spring were the main reason behind supplies flow of Egyptians' gas to Jordan, which negatively affected the energy bill. As a result, the gross national product's growth rate contracted from 7.9% in 2008 to 2.3 in 2010. This contraction was accompanied by adverse effects such as unemployment rates and an increase in essential commodities' prices (Carnegie endowment for international peace, 2015, Carnegie Middle East, 2015).

Over the nine years of the Syrian humanitarian presence in Jordan, the asylum's economic impact has become realistic and constitutes an official and widespread concern. Giving that asylum has affected both Syrian refugees and vulnerable Jordanians' food security and livelihood. The Syrian asylum has affected Jordan's social and economic fabric (MOPIC, 2020). Jordan issued work permits for refugees, whose numbers reached 179,445 for the period between 2016–20, resulting in economic effects on the Jordanian labor market (Ministry of Labor, 2020). Giving that Syrian workers compete with Jordanians coincide with sectors' desire to hire Syrians who do not insist on high salaries, health insurance, or social security benefits. According to the Ministry of Labor's statistics, the total unemployment rates before the crisis were 14% in 2019 it reached 19% (Jordanian Department of Statistics, 2019). The 2020 JRP stressed that Jordan faces food security's challenges. The plan indicated that Jordan recorded an average of 10.5 in World's Hunger Index for 2019 compared to 6.7 in 2017; this rate is annually increasing significantly. According to the plan, the poverty rate reached 17.9 in 2020 compared to 14.4 in 2010, which is an alarming and worrying indicator of the Jordanian food security situation (MOPIC, 2020). The increase in poverty rates combined with the high hunger index is evidence of pressure Jordan faces in securing food for Syrian refugees, whether inside or outside the camps.

The scarcity of water resources is among the permanent challenges Jordan has always faced. The Jordan river used to supply Jordan with most of its water needs. However, Israel's control of the river prevented Jordan's share of water despite the regulation of water shares between Jordan and Israel according to the 1994 peace agreement. Nowadays, Jordan is the second poorest country globally in terms of water availability (Ministry of Water and Irrigation National Water Strategy, 2016).

As a result of the Syrian refugee influx into Jordan, which caused a massive and sudden population increase, the water supply was affected. The annual water demand increased significantly in the Northern Governorates. The increase rate was 40%. Since the Syrian crisis, most of the renewable surface water is used simultaneously, and groundwater is exploited in an unsustainable manner, which inevitably leads to a decrease in groundwater level and a deterioration in its quality. This also coincided with the lack of sanitation networks in Northern governorates where the refugees reside in camps, resulting in the possibility of contaminating the groundwater (Ministry of Water and Irrigation National Water Strategy, 2016). The increase in demand for water has led to tremendous pressure on water resources and negatively affects Jordan's ambitions to achieve sustainable development. Climate change and its adverse effects on the scarcity of surface and underground water resources whose rates will decrease 15% by 2040. This situation may constitute a security threat to Jordan's existence, stability, and survival.

### 4.5 UNHCR, the donor states, and international Community's responsibilities towards Jordan

The King of Jordan in 2016 warned that Jordan would permanently stop receiving Syrian refugees if the international community did not adhere to its responsibilities towards helping the Jordanian efforts and stressed that the dam would ' burst' (BBC, 2016). The King emphasized that Jordanians are " at boiling point", and the international community should act immediately. He further added, during his interview with BBC correspondent, that Jordan has reached a 'boiling point' (BBC, 2016). He noted that only several thousand Syrian children have received educational opportunities and that hospitals and chances to gain jobs among Syrians suffer from unbearable pressure. In his conversation, the King pointed out that the UN had asked Jordan to receive the refugees and pledged that UNHCR, donor countries, and the international community would help Jordan in its humanitarian efforts. The UN has affirmed that rich countries will adhere to the fair- share principles in donating money and assisting in marinating refugees' camps. However, according to the King, since the beginning of the crisis, the international community failed to fulfill its duties, as required and agreed. This requires the international community to fulfill the promise according to goodwill rule, as confirmed by the king (BBC, 2016).

For the second time, the King indicated during the World Economic Forum Davos Agenda 2021 – held via teleconference; that protecting refugees is an international responsibility, especially during the Coronavirus Pandemic. During the speech, the King stressed that Jordan is the second country in the World to receive humanitarian asylum per capita. He pointed out that Jordan is still committed to welcoming and caring for refugees even within the exceptional economic circumstances Jordan is going through resulting from the pandemic's repercussions. In this matter, the King emphasized that the challenges that face Jordan during the pandemic are outstanding. He adds, " amid these challenging times, safeguarding the health and well-being of refugees remains a global responsibility" (King Abdallah Official Website, 2021). He further pointed out that Jordan was one of the first countries to provide the vaccine to refugees for free. According to him, this requires the international community's need to help Jordan in its efforts to control the epidemic among camp's refugees. Meanwhile, stressing that global support is at its lowest levels. Hence, more support is required, which is necessary so that Jordan collectively, can provide care for refugees and receive more of them, or resorting to closed borders' strategy, which may result in humanitarian catastrophe (King Abdullah Official Website, 2021).

In his speech delivered at the leaders' Summit on Refugees- on the margins of the 71 UN General Assembly, King Abdallah (2016) indicated that " the refugee crisis requires not just commitment but follow-through." He further stressed that the refugee crisis requires " immediate collective responsibility and global engagement without delay" (King Abdullah Official Website, 2016).

In the same vein, the Jordanian Interior Minister in 2020 called on donor countries to provide adequate support to assist JRP's implementation. This assistance benefits Syrian refugees in Jordan (MSN News, 2020). The Jordanian RP for years 2020–22 indicates that Jordan remains committed to its moral obligations towards Syrian humanitarian asylum. Since 2012, Jordan bears more than its fair share of accommodating short and long-term needs for Syrian refugees, which have exhausted Jordan's absorptive capacity (MOPIC, 2020). Jordan does not deny UNHCR, donor countries, and the international community's support during the years of Syrian refugees' asylum, however according to JRP. This support is modest, shy, scarce and, it is inconsistent with the heavy responsibilities that fall on Jordan. During the years of asylum, Jordan suffered from the Syrian asylum's consequences. However, Jordan fulfilled its international moral obligations. Against this, Jordan cannot perform its humanitarian duties alone, giving tremendous economic pressure, scarcity of resources, and infrastructure depletion, requiring the urgent need for international efforts to unite through real support, and financing. The Jordanian RP further pointed out that the support Jordan received from the UNHCR, donor countries, and the international community did not exceed 51% of the requirements of JRP for the year 2019, and the rest is financially supported by Jordan alone. The 2020 JRP, in its preamble, stresses Jordanian seriousness in responding to the Syrian humanitarian asylum and its commitment to the 2030 international plan that resulted from Brussel's I, II, and III conferences of donor countries (MOPIC, 2020). Therefore, according to JRP, the Jordanian response plan is the only one capable of implementing what was agreed on in the international conferences regarding the Syrian refugees. Hence, the program requires real international support to implement its provisions on the ground (MOPIC, 2020).

For Jordan to meet Syrian refugees' asylum needs and fulfill its Humanitarian responsibilities for the years 2020–22, according to JRP, a total budget of USD 6,607,129,404 is required (MOPIC, 2020). This budget covers the following sectors: public services, health, education, water supply and management, economic empowerment, food security and livelihood, social and judicial justice protection (MOPIC, 2020). According to Jordanian officials, the Syrian asylum crisis has come when the country suffers from enormous economic challenges that have critically harmed social, economic, institutional, and national resources (Jordanian Economic and Social Council, 2015). The direct estimates of Syrian refugees' negative impact on Jordan are very formidable. The country bears an excellent part of the crisis's repercussions in light of the limited aid it receives from the international community. The direct effects of the Syrian refugees' crisis include the monetary impact on the state's budget. The Real Gross Domestic Product (RGDP) decreased from 5.5% during 2006–11 to 2.4% during 2012–18 (MOPIC, 2020). Simultaneously, the unemployment rate jumped from 12.2% in 2012 to 19.1% in 2019. In the same context, total domestic and external public debt, as a percent of GDP, increased from 67.4% for years 2006–11 to 96.9% in 2020 (MOPIC, 2020), and this is a worrying percentage, giving that domestic income is equivalent to public debt and hence leaving nothing to sustain the state's survival. According to General Budget Department, the public debt in 2010 did not exceed USD 20 Billion. However, it reached USD 50 Billion at the end of the 2020 fiscal year, which constitutes 98.2% of the Jordanian GDP (General Budget Department, 2020).

During the end of 2020, UNHCR called on all signatory states to the 1951 refugee convention and donor states to fulfill their financial pledges to achieve its relief and humanitarian obligations. Meanwhile, the Commissioner announced that it had received USD 4.5 Billion out of 9.1 Billion, which is the total funding amount agreed upon (Petra Jordanian News Agency, 2020). The Commissioner also indicated that it only received in 2020 approximately half of the amount, which according to it, will negatively affect the lives of millions of refugees and also hosting states. Giving that UNHCR's scope of its relief and humanitarian operations includes many regions in the world. The surprising and repulsive issue, which UNHCR reported, is that non-fulfillment of obligations and ignoring responsibilities' sharing is a chronic phenomenon. This has forced UNHCR to either reduce or stop some of its relief and humanitarian activities in host states, including food and other aid (Petra Jordanian News Agency, 2020). According to Washington Institute (2016), King Abdullah's remarks on Jordan's Syrian refugees' repercussions are not exaggerated. The institution further shows that Jordan is in dire need of additional aid, giving that Syrian refugees constitute a financial burden that Jordan, which suffers from the scarcity of resources and high debt, cannot do it alone. This calls on UNHCR, donor countries, and the international community to meet their financial commitments (The Washington Institute for Near East Policy, 2016).

At the end of 2019, Jordanian foreign affairs Minister Ayman Safadi emphasized that asylum's burden is a shared international community's responsibility and cannot be borne on by host states alone. During the World Refugee Forum's session held in Geneva in 2019 under the title" burden-sharing arrangements." Safadi stressed that international collective action is an urgent necessity to meet the refugees needs. According to him, refugees' issues require sharing responsibility through providing financial and material assistance and sustainable programs (Safadi, 2019). Safadi indicated that investing in refugees by providing their needs that secure a decent life is a real investment in common international collective security. Giving that, marginalization of refugees through exposing them to despair, ignorance, poverty, and deprivation will provide an appropriate for the growth of radicalism, extremism, fanaticism, and negativity (Safadi, 2019).

In this regard, one might cite Baqa'a, Rukban, and Karak's terrorists' attacks in 2016, which resulted from exporting ISIS's terrorist ideology to Jordan. The terrorist network based in Syria and many of its followers entered Jordan as refugees. In those three attacks, 20 individuals lost their lives, and many were severely injured. Investigations revealed that the attack perpetrators were Jordanians whom ISIS's ideology has influenced them. Also, the investigations found out that those terrorists have been communicating with ISIS's followers, who were Syrian refugees living either inside or outside the camps. The attackers were directed by those refugees' plans to conduct attacks against Jordanian security agencies. Meanwhile, the attackers indicated that their attacks were executed due to the Jordanian closed-borders policy adopted in the years 2014–15 (Ammon News, 2016).

Syrian refugees' continued presence in Jordan could be endangered by various elements such as (1) the UNHCR's inability to fulfill its obligations towards Syrian refugees in Jordan, (2) the 1951 convention signatory states' unwillingness to adhere, entirely, to their financial responsibilities, (3) lack of donor countries' seriousness, and (4) the international community's failure to fulfill their obligations towards Jordan. It is known that Jordan is a developing country with limited resources and a crumbling economy. Jordan, for the longest, suffers from structural financial dilemmas. In light of continuing depletion of the low Jordanian budget and lack of international assistance needed to sustain humanitarian efforts, Syrian asylum in Jordan may be threatened with discontinuation. Among possible scenarios for Jordan is to adopt closed- borders policy that has been implemented in the years 2014–15. If adopted, this policy would inevitably endanger displaced Syrian who wish to cross Jordanian borders seeking safety and shelter.

The second possible scenario is Jordan resorting to refoulment under the pretext of financial inability to maintain their presence. Through its 2020's RP, Jordan indicated the subtotal amount needed to sustain Syrian refugees' existence in Jordan for years 2020–22. Accordingly, failure to fulfill donor countries and the international community's commitments may negatively affect Syrian refugees in Jordan. It is known that Jordan is not a signatory of the 1951 refugees' convention or its 1967 protocol. When Jordan signed the Memorandum of Understating with UNHCR in 1998, it was agreed upon that the Commission must adhere to all its responsibilities towards Jordan, and failure to fulfill the obligation on the part of UNHCR will result in Jordan not being committed to the memorandum's provisions. Therefore, donor countries and the international community must act urgently to avoid unfortunate consequences on refugees, which may endanger their lives if they forcibly deported or close the borders in front of them and thus prevent the reception of more persecuted people fleeing oppression and inhuman treatments.

Jordan, based on what was mentioned above, and in light of the scarcity of the financial resources necessary to sustain the Syrian humanitarian asylum and meanwhile receive more displaced refugees, does not have a magic wand. It is universally known that the implementation of humanitarian refugee policies on the ground requires logistical and multiple and permanent financial sources, and this exceeds Jordan's ability and capabilities. What is hence deemed necessary is concerted international serious efforts to help Jordan overcome obstacles that may inevitably affect the presence of Syrian refugees in Jordan and at the same time may negatively affect the quality of services and facilities provided to them. At present, Jordanian are consenting on a central point, which is that Syrian asylum has become burdensome on the state's budget, which requires rapid, serious, and effective financial intervention from the UNHCR, donor countries, neighboring oil-rich countries, and the international community as a whole. Otherwise, the results will be dire and may reflect on the security and stability of the region which may threaten international peace and security.

## 5 Conclusions and recommendations

Jordan shares long border with Syria, which is the main reason behind the Syrian refugees' influx towards adjacent areas. Over the long stages of its political existence, Jordan received frequent and intense waves of refugees. The Palestinian asylum waves of 1948 and 1967, and in later stages, the return of Palestinian from Kuwait due to Kuwait's Iraqi invasion. In addition to Iraqi asylum in years 1991 and 2003 and the last of which was Syrian humanitarian influx into Jordan. Jordan dealt with these humanitarian waves according to its concrete moral commitments. Jordan is not a signatory of the 1951 Refugee Convention or its Optional Protocol giving the deep Jordanian belief that Palestinians have the inherent right to return to their homeland that the Zionists had occupied in 1948. Jordan's desire to contribute to international humanitarian efforts drove it to sign the Memorandum of Understanding with UNHCR in 1998 to carry out its humanitarian activities and duties in Jordan.

The Jordanian Constitution prohibits the refoulment of political refugees, and the same applies to any refugee entering the Kingdom. Jordan is also committed to the 1994 Arab Charter on Human Rights, which is entirely consistent with the 1951 refugee convention. It is worth noting that the legal system in Jordan has only dealt with diplomatic asylum. Meanwhile, the Jordanian constitution has not addressed, mentioned, or dealt with the rest of other asylum types including humanitarian asylum. However, Jordan's signing of the Memorandum of Understanding with UNHCR in 1998 indicates - in terms of legal and objective terms, Jordan's commitment to the 1951 Refugees' Convention, and the Arab Charter of Human Rights. Despite this, the Jordanian constitutional texts were not amended to deal with Jordan's commitment to receiving humanitarian asylum. However, the legislative authority's approval of the 1998 Memorandum of Understanding legally means Jordan's commitment to receiving all types of refugees. In Jordan, the rules of international common law's treaties and rules transcend internal law and become enforceable and binding on all authorities after ratification by legislature authority.

The Jordanian Ministry of Interior and other Ministries are entrusting with supervising refugees' statues through documenting their entry into the Kingdom. The Ministry cooperates with UNHCR to determine the refugee's eligibility to enter and secure shelter and care. To this end, the Directorate of Syrian Refugees' Affairs was established in 2014 to deal with Syrian humanitarian asylum issues in Jordan. Meanwhile, in 2014 authorities were given to Jordan Hashemite Charity Organization to receive local and international assistance and donations and distribute them to Syrian refugees. For Jordan to systematically deal with Syrian refugees, the Jordanian Response Plan (JRP) was introduced in 2015. The program was rolled over for years 2016–18, and then the current plan for years 2020–22. In these plans, refugees' needs, including education, health, aid and food, services, water, shelter, logistics, and security sectors, are detailed, financially. Jordan does not deny UNHCR, donor states, or the international community endless efforts nor the assistance it receives from them; however, the presence of Syrian refugees has exhausted the budget and the scarce economy. Hence, the international community must financially assist Jordan in implementing the current response plan on the ground.

The lack of Jordan's financial resources for Jordan may negatively affect Syrian refugees, and Jordan may resort to a closed-borders policy or refoulment. These measures are not desirable for Jordanian people; however, economic burdens may drive Jordan towards unpleasant consequences. This point of view has been emphasized by King Abdullah, who warned that the dam would ' burst' any time and that Jordan's refugees' hosting got to a boiling point. The King delivered an important message that entailed Jordan's inability to embrace the refugees alone. Failure to receive the supposed aid may deepen extremist and radical thinking among Syrian refugees. It is recommended that UNHCR, donor states, and international community assist Jordan in its efforts to continue hosting Syrian refugees and to welcome more if they were forced to flee Syria searching for peace and security.

## Declarations

### Author contribution statement

Amir Salameh Al Qaralleh: Conceived and designed the experiments; Performed the experiments; Analyzed and interpreted the data; Wrote the paper.

### Funding statement

This research did not receive any specific grant from funding agencies in the public, commercial, or not-for-profit sectors.

### Data availability statement

Data included in article/supplementary material/referenced in article.

### Declaration of interests statement

The authors declare no conflict of interest.

### Additional information

No additional information is available for this paper.
